# Should Blood Cultures Be Drawn Through an Indwelling Catheter?

**DOI:** 10.1093/ofid/ofae248

**Published:** 2024-05-02

**Authors:** Leonard A Mermel, Mark E Rupp

**Affiliations:** Division of Infectious Diseases, Department of Medicine, Warren Alpert Medical School of Brown University, Providence, Rhode Island, USA; Department of Epidemiology and Infection Prevention, Lifespan Hospital System, Providence, Rhode Island, USA; Division of Infectious Diseases, University of Nebraska Medical Center, Omaha, Nebraska, USA

**Keywords:** bacteremia, blood culture, bloodstream infection, catheter infection, central line associated bloodstream infection

## Abstract

There is no practical way to definitively diagnose a catheter-related bloodstream infection in situ if blood cultures are only obtained percutaneously unless there is the rare occurrence of purulent drainage from a central venous catheter insertion site. That is why the Infectious Diseases Society of America guidelines for diagnosis and management of catheter-related bloodstream infections and Infectious Diseases Society of America guidelines for evaluation of fever in critically ill patients both recommend drawing blood cultures from a central venous catheter and percutaneously if the catheter is a suspected source of infection. However, central venous catheter–drawn blood cultures may be more likely to be positive reflecting catheter hub, connector, or intraluminal colonization, and many hospitals in the United States discourage blood culture collection from catheters in an effort to reduce reporting of central-line associated bloodstream infections to the Centers for Disease Control and Prevention. As such, clinical decisions are made regarding catheter removal or other therapeutic interventions based on incomplete and potentially inaccurate data. We urge clinicians to obtain catheter-drawn blood cultures when the catheter may be the source of suspected infection.

## MAIN POINT

Blood cultures should be collected from a central venous catheter and percutaneously when the catheter is a possible source of infection.

Clinicians in many US hospitals are discouraged from obtaining blood cultures from indwelling central venous catheters (CVCs; personal communication) to reduce the likelihood of positive blood cultures resulting from catheter colonization, which may lead to reporting a central line-associated bloodstream infection to the NHSN surveillance system. The motivation behind this change in practice reflects the fact that central line-associated bloodstream infections are associated with potential loss of hospital reimbursement from the Centers for Medicare & Medicaid Services and third-party payers, as well as potential damage to institutional reputation. However, this practice is based on older studies [1, 2] before the increasing use of port protectors [3, 4] and conflicts with Infectious Diseases Society of America guidelines [5, 6]. Additionally, how can a clinician definitively diagnose a catheter-related bloodstream infection (CRBSI) if only percutaneously drawn cultures are obtained unless the patient has purulent drainage from the insertion site (uncommon in patients with infected CVCs; more common with infected short-term peripheral venous catheters), or the catheter is removed and the tip is cultured revealing the same microbe as in the blood? It is also important to remember that CRBSI reflects a dynamic process. Bacteremia or fungemia emanating from a colonized catheter may be intermittent depending on whether or not fluid has been infusing through a colonized catheter lumen, the type of infusate (eg, an antibiotic infused through the catheter lumen just before blood draw may lead to false-negative blood cultures), characteristics of the colonizing microorganism and density of intraluminal microbial growth [7, 8], as well as the immune status of the patient (eg, does the patient have a functional liver and spleen to clear pathogens from the bloodstream) [8]. When fluids are flowing through a colonized catheter, patients may develop fever or other signs of systemic infection as microbes are pushed into the bloodstream. During such times, peripheral blood cultures may be positive. A patient's symptoms may resolve when the fluids are no longer being infused through the catheter and percutaneously drawn blood cultures may be negative. This scenario is most evident in patients receiving hemodialysis through a central venous catheter who become unwell during dialysis but whose symptoms improve after completion of dialysis.

A positive catheter-drawn blood culture in the absence of growth from a percutaneously drawn culture may reflect contamination, especially with growth of common skin commensals, or a greater volume of blood drawn from catheters for culture compared with blood cultures drawn from peripheral veins [9]. This finding should not be disregarded as contamination in all cases, particularly with growth of microbes commonly causing serious bloodstream infections (eg, *Staphylococcus aureus*), or hemodynamically unstable patients without an alternative explanation for their symptomatology. Repeat blood cultures may be indicated with continued symptoms without a clear source. Additionally, in patients with long-term catheters, this finding may be a signal that the catheter is colonized and clinicians may consider catheter lock therapy in an effort to eradicate colonization of the catheter because studies have demonstrated that without intervention, such patients may go on to have microbial growth from percutaneously drawn cultures over the ensuing weeks [10]. When there is growth from both catheter-drawn and percutaneously drawn blood cultures, a differential time to blood culture positivity may assist in identifying the catheter as the source of the bloodstream infection [11]. On the other hand, the negative consequences of blood culture contamination should not be dismissed as blood culture contamination is clearly associated with extended length of hospital stay, inappropriate antibiotic use, and diagnostic confusion [12].

When encountering a patient with possible CRBSI, a pertinent question is how many lumens should be sampled if the CVC is thought to be a likely source of infection. This is important because many critically ill patients with fever or sepsis have multiple intravascular catheters, often with multiple lumens. Sampling each lumen could result in many blood cultures leading to increased cost, iatrogenic anemia, and additional opportunities for contamination. However, approximately one-third of CRBSIs will be missed if 1 lumen of a multilumen catheter is sampled [13]. Despite controversy regarding which lumen and how many lumens should be sampled for blood culture collection, the lumen used for administration of total parenteral nutrition and/or blood products may have the highest yield [14]. Initially performing only peripheral blood cultures in patients with suspected sepsis and then going back to obtain catheter-drawn cultures to establish the catheter as the source has substantial downsides. First, after obtaining initial blood cultures, in many instances, empiric antimicrobial therapy will be initiated, making subsequent cultures from the catheter less reliable. Second, delay in diagnosis of CRBSI could result in a delay in removal of an infected catheter with poor source control and greater chance for metastatic spread and poor outcome. Accurate assessment of which lumen(s) are involved is particularly important if an attempt at catheter salvage with catheter lock therapy is contemplated.

## RECOMMENDATIONS

- Percutaneously drawn blood cultures should be obtained when blood cultures are indicated.
- Blood cultures should be drawn from a catheter if there is reasonable clinical suspicion that the catheter could be the source of infection: there is evidence of localized infection (eg, purulent drainage, suspected tunnel infection); fever, and/or hypotension during or shortly after infusion through a catheter, or without obvious source based on careful assessment of the patient; unexplained change in a patient's status during hemodialysis; or if one simply cannot obtain blood cultures percutaneously.
- Catheter-drawn blood cultures should be accompanied by percutaneously drawn cultures whenever possible.
- Avoid catheter-drawn blood cultures if a nonvascular catheter source of infection is likely.
- Because a catheter is unlikely to be the source of infection when in situ less than 48 hours, catheter-drawn blood cultures should be minimized in such scenarios unless the catheter was inserted under emergent conditions with possible breach in aseptic technique (eg, femoral CVC placed during a code situation) and no other source of infection is identified.
- Catheter-drawn blood culture contamination can be minimized by using care to reduce risk of contamination by removal of existing connector valves, disinfection of the catheter hubs, and obtaining the blood through a fresh sterile connector or disinfected hub. Additionally, passive port protectors should be used more widely to prevent catheter colonization and blood culture contamination.
- In patients with a multilumen CVC, more than 1 lumen should be sampled. However, the clinician must balance the likelihood of CRBSI and the need to sample multiple lumens against the downsides of increased cost, iatrogenic anemia, and increased risk of contamination.
- Because approximately 90% of blood cultures are without growth, blood culture diagnostic stewardship programs should be employed to avoid blood cultures with low pretest probability [15, 16].

In sum, patient who have evidence of other sites of infection for whom blood cultures are obtained (eg, patients with a dehisced surgical wound with purulent drainage, patients with dysuria and flank pain), drawing all blood cultures percutaneously makes sense. However, in patients with CVCs whose clinical status suggests an infection, but the source is not evident on history and physical examination, then a catheter infection is in the differential diagnosis and blood cultures should be obtained from the catheter and percutaneously. We should strive for better surveillance definitions that do not inadvertently dissuade clinicians from delivering best care and do not inappropriately penalize institutions. We recommend that national surveillance programs analyze separately blood cultures growing common skin commensals, even if drawn on 2 separate occasions. Better methods to prevent CVC colonization and CRBSI are needed, along with more widespread application of proven technologies to prevent CRBSI. Application of proven interventions to minimize blood culture contamination should be used when blood cultures are performed. Better means to detect CVC colonization and CRBSI while the CVC is in situ would be very helpful. Last, studies are needed comparing blood culture contamination using a blood culture diversion device [17] for percutaneously drawn cultures and blood culture contamination when drawn through a central venous catheter hub after removal of a needleless connector and antiseptic barrier cap [3].

## Notes

***Acknowledgments.*** There was no funding for this work.
